# High frequency oscillatory ventilation and prone positioning in a porcine model of lavage-induced acute lung injury

**DOI:** 10.1186/1471-2253-6-4

**Published:** 2006-04-03

**Authors:** Joerg Brederlau, Ralf Muellenbach, Markus Kredel, Clemens Greim, Norbert Roewer

**Affiliations:** 1 Klinik und Poliklinik für Anästhesiologie, Universitätsklinikum Würzburg, Oberdürrbacher Str. 6, 97080 Würzburg, Germany; 2 Klinik für Anästhesiologie, Intensiv- und Notfallmedizin, Klinikum Fulda, Pacelliallee 4, 36043 Fulda, Germany

## Abstract

**Background:**

This animal study was conducted to assess the combined effects of high frequency oscillatory ventilation (HFOV) and prone positioning on pulmonary gas exchange and hemodynamics.

**Methods:**

Saline lung lavage was performed in 14 healthy pigs (54 ± 3.1 kg, mean ± SD) until the arterial oxygen partial pressure (PaO_2_) decreased to 55 ± 7 mmHg. The animals were ventilated in the pressure controlled mode (PCV) with a positive endexpiratory pressure (PEEP) of 5 cmH_2_O and a tidal volume (V_T_) of 6 ml/kg body weight. After a stabilisation period of 60 minutes, the animals were randomly assigned to 2 groups. Group 1: HFOV in supine position; group 2: HFOV in prone position. After evaluation of prone positioning in group 2, the mean airway pressure (P_mean_) was increased by 3 cmH_2_O from 16 to 34 cmH_2_O every 20 minutes in both groups accompanied by measurements of respiratory and hemodynamic variables. Finally all animals were ventilated supine with PCV, PEEP = 5 cm H_2_O, V_T _= 6 ml/kg.

**Results:**

Combination of HFOV with prone positioning improves oxygenation and results in normalisation of cardiac output and considerable reduction of pulmonary shunt fraction at a significant (p < 0.05) lower P_mean _than HFOV and supine positioning.

**Conclusion:**

If ventilator induced lung injury is ameliorated by a lower P_mean_, a combined treatment approach using HFOV and prone positioning might result in further lung protection.

## Background

Prevention of irreversible hypoxemic damage and improvement of respiratory mechanics are the main treatment goals in patients with acute respiratory distress syndrome (ARDS). Mechanical ventilation is the predominant supportive treatment modality in ARDS, but has also detrimental side effects, currently termed ventilator induced lung injury (VILI).

Lung protective ventilation strategies aim to ameliorate VILI by application of a reduced tidal volume (V_T _= 6 ml/kg), sufficient PEEP-level and limitation of the plateau inspiratory pressure to 35 cm H_2_O [1-3].

Although it is known, that the degree of hypoxemia is inconclusive to predict mortality [4], the early response of the PaO_2_/FIO_2_-ratio to therapeutic interventions might be an indicator for an increased survival rate in ARDS [5,6]. This calls for the most rapid amelioration of hypoxemia with a mono- or multimodal organ protective treatment approach.

High frequency oscillatory ventilation (HFOV) with its constant mean airway pressure (P_mean_) with superimposed small tidal volumes and active in- and expiration at a high respiratory frequency might be the ideal lung protective ventilatory strategy [7]. In a multicenter randomized controlled trial investigating the effectiveness of HFOV, the significant early improvement of the PaO_2_/FIO_2_-ratio in the HFOV-group was associated with a tendency towards a reduced 30-day mortality compared with the conventional ventilation group. The PaO_2_/FIO_2_-ratio was the most significant predictor of survival independent of the selected ventilator strategy [8].

Prone positioning was shown to increase the PaO_2 _in 70–80% of patients with ARDS and to improve alveolar ventilation without influencing the 28-day mortality [9,10]. If PaCO_2_-reduction was achievable with prone positioning, 28-day mortality in ARDS patients was significantly reduced [11]. Prone positioning and application of PEEP were shown to have an additive effect on oxygenation [12]. However, prone positioning is a potentially dangerous manoeuvre with acute and long term complications such as tracheal tube dislocation, and pressure sores [13].

Combination of different treatments are used in desperation for salvage therapy in patients with ARDS [14]. A recently published study in 39 medical ARDS-patients randomized to conventional lung protective ventilation and HFOV showed comparable increases of the PaO_2_/FIO_2_-ratio after prone positioning. An additive effect of prone positioning and HFOV could not be demonstrated [15].

The objective of our study was to evaluate the effects of prone positioning on gas-exchange, hemodynamics and respiratory parameters in HFOV-ventilated pigs with severe lavage induced acute lung injury [16]. We hypothesized, that during HFOV oxygenation can be improved at a lower P_mean _with the animals positioned prone than supine.

## Methods

### Animals

The study was conducted in accordance with the National Institutes of Health guidelines for ethical animal research and was approved by the Laboratory Animal Care and Use Committee of the District of Unterfranken, Germany.

The experiments were performed in 14 healthy pigs, Pietrain breed, all negative for the malignant hyperthermia gene. The animals were 14 to 18 weeks old, with a mean (± SD) body weight of 54 ± 3.1 kg.

### Experimental preparation

The animals were fasted for 24 hours without limiting water access. Prior to instrumentation the animals were sedated with intramuscular ketamin (10 mg/kg), xylazine hydrochloride (1 mg/kg) and atropine (25 μg/kg) and placed supine on an operating table armed with a heating pad to provide core temperature stability (37.3 ± 0.5°C). Anesthesia was induced with an intravenous bolus of sodium thiopental (5 mg/kg) using an auricular vein. The animals' trachea was orally intubated with a cuffed 8.0-mm ID Edgar tracheal tube (Rueschelit^®^, Ruesch, Kernen, Germany) providing an additional lumen embedded in the tubes inner wall for tracheal pressure monitoring. Anesthesia and complete muscle relaxation were maintained with continuous intravenous infusion of ketamin (2 mg/kg/h), midazolam (0.5 mg/kg/h), fentanyl (0.01 mg/kg/h) and vecuronium (0.1 mg/kg/h).

The animals were mechanically ventilated with a Servo^® ^900C ventilator (Siemens-Elema AB, Solna, Sweden) using pressure controlled ventilation (PCV) with a PEEP of 5 cmH_2_O, an inspiratory to expiratory ratio (I:E) of 1:1 and a fraction of inspired oxygen (FIO_2_) of 1.0. A V_T _of 6 ml/kg and a respiratory rate (RR) of 25–30 breath/min were applied resulting in normocapnia.

After a bolus of 500 ml balanced electrolyte solution a continuous infusion was given at a rate of 2–6 ml/kg/h. Continuous electrocardiography (Servomed^®^, Hellige, Freiburg i. Br., Germany), pulsoxymetry, capnography and distal tracheal pressure monitoring (SM8050^®^, Draeger, Luebeck, Germany) were performed.

2 gm Cefotiam was administered intravenously. After systemic heparinization (300 U/kg Liquemin^®^, Roche, Reinach, Switzerland) arterial and central venous access were established transcutanuously using ultrasound guidance (SonoSite 180 Plus^®^, SonoSite Inc., Botell, WA, USA). Activated clotting time (ACT II^®^, Medtronic, Minneapolis, MN, USA) was measured hourly and maintained between 150 and 200 seconds throughout the experiment with heparin bolus injections as needed. The left carotid artery was cannulated with a 20-gauge catheter (Vygon, Ecouen, France). The right internal jugular vein was cannulated with a 9 French introducer sheath (Arrow, Reading, PA, USA) and a 7,5 French flow directed thermodilution pulmonary artery catheter (831F75, Edwards Lifescience, Irvine, CA, USA) was advanced into the pulmonary artery under transduced pressure guidance.

### Hemodynamic, ventilatory and blood gas measurements

For hemodynamic monitoring pressure transducers referenced to atmospheric pressure at the mid-thoracic level (Combitrans^®^, Braun, Melsungen, Germany) and a modular monitor system (Servomed^®^, Hellige, Freiburg i. Br., Germany) were used. Mean arterial pressure (MAP), mean pulmonary artery pressure (MPAP), central venous pressure (CVP) and pulmonary artery occlusion pressure (PCWP) were transduced. Heart rate (HR) was traced by the electrocardiogram.

Trifold injections of 10-ml aliquots of ice cold saline into the right atrium at random phases of different respiratory cycles were used for pulmonary artery catheter-based cardiac output (CO)-measurements (Explorer^®^, Edwards Lifescience, Irvine, CA, USA).

Arterial and mixed venous blood samples were immediately analyzed for PO_2_, PCO_2 _and pH using standard blood gas electrodes (ABL 505^®^, Radiometer, Bronshoj, Denmark). In each sample, hemoglobin and oxygen saturation were measured using spectrophotometry (OSM3^®^, Radiometer, Bronshoj, Denmark). Arterial (CaO_2_), mixed venous (CvO_2_) and pulmonary capillary (CCO_2_) oxygen contents (ml/dl) and the pulmonary shunt fraction (Qs/Qt) were calculated using standard formulas. The oxygenation index (OI) was calculated using the formula introduced by Hallmann et al.: OI = (P_mean _× FiO_2 _× 100) / PaO_2 _[17].

For tracheal pressure monitoring air filled pressure transducers (Combitrans^®^, Braun, Melsungen, Germany) referenced to atmospheric pressure were used [18]. Temperature was measured by thermistor in the pulmonary artery.

### Experimental procedure

#### Lung injury

After instrumentation the animals were stabilized for 30 min in the supine position and mechanically ventilated with PCV (V_T _= 6 ml/kg, I:E = 1:1, FIO_2 _= 1.0, PEEP = 5 cmH_2_O). RR was adjusted to achieve normocapnia. Baseline measurements were obtained.

Lung injury was induced by bilateral pulmonary lavages with 30 ml/kg isotonic saline (38°C) and repeated every 10 minutes until PaO_2 _decreased to 40–60 mmHg and was stable for 60 minutes with unchanged ventilator parameters. During induction of lung injury all lungs were ventilated with PCV, FIO_2 _= 1.0, PEEP = 5 cmH_2_O, V_T _= 6 ml/kg, RR = 40/min. Post injury measurements were obtained.

### Positioning

Prone positioning was performed with supportive rolls under shoulders and pelvis providing a free abdomen in order to minimized increases in intra-abdominal pressure.

### Study protocol

The FIO_2 _(1.0) remained unchanged throughout the experiment. A 20-min equilibration period was given for each modification following the study protocol. After time point T_0 _the standard ventilator was replaced by an oscillatory ventilator (Sensormedics 3100B, Viasys, Conshohocken, PA, USA) without changes in P_mean_. The animals were randomly assigned to two groups (n = 7 each):

Group 1 : HFOV (Bias flow = 30 l/min, amplitude = 70 cm H_2_O, I:E = 1:1, RR = 300/min).

Group 2 : HFOV (Bias flow = 30 l/min, amplitude = 70 cm H_2_O, I:E = 1:1, RR = 300/min) and prone positioning.

**Figure 1:**
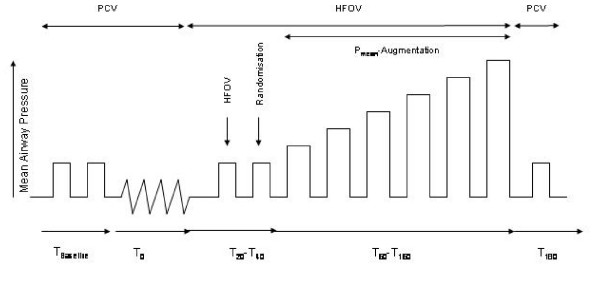
Experimental protocol T_baseline_: 30 min after instrumentation. T_0_: 60 min after last pulmonary lavage. T_20_: HFOV (P_mean _= 16 cmH_2_O). T_40_: prone positioning in group 2. T_60_: P_mean _increased from 16 to 19 cmH_2_O. T_80_: P_mean _= 22 cmH_2_O. T_100_: P_mean _= 25 cmH_2_O. T_120_: P_mean _= 28 cmH_2_O. T_140_: P_mean _= 31 cmH_2_O. T_160_: P_mean _= 34 cmH_2_O. T_180_: P_mean _= 16 cmH_2_O (≈ T_0_) PCV in all groups with HFOV and prone positioning discontinued.

A 20-min period was given for equilibration between each modification and followed by measurements of hemodynamics, blood gases and respiratory parameters. The following modifications were performed after completion of measurements terminating the previous 20-min period (Figure 1):

(1) T_baseline_: 30 min after instrumentation.

(2) T_0_: 60 min after last pulmonary lavage.

(3) T_20_: HFOV (P_mean _= 16 cmH_2_O) had been started in both groups

(4) T_40_: The animals had been positioned prone in group 2; no changes were made in group1.

(5) T_60_: P_mean _had been increased from 16 to 19 cmH_2_O. P_mean_, measured at the tip of the endotracheal tube, was the ventilatory parameter modified during the experiment from time point T_40 _onwards. To change P_mean_, continuous distending pressure (CDP) was elevated in steps of 3 cm H_2_O.

(6) T_80_: P_mean _= 22 cmH_2_O. (7) T_100_: P_mean _= 25 cmH_2_O. (8) T_120_: P_mean _= 28 cmH_2_O. (9) T_140_: P_mean _= 31 cmH_2_O. (10) T_160_: P_mean _= 34 cmH_2_O.

(11) T_180_: P_mean _had been decreased to 16 cmH_2_O (≈ T_0_). HFOV had been discontinued and ventilation had been set to T_0_-values in all groups (PCV, PEEP = 5 cm H_2_O, V_T _= 6 ml/kg, RR = 40/min). The animals in group 2 had been positioned supine.

At the end of the experiment the animals were killed using an intravenous overdose of sodium thiopental and T 61 (Intervet, Unterschleissheim, Germany).

### Statistical analysis

Values are reported as mean ± SD. Statistical analyses were performed with Statistica for Windows, version 5.1 (StatSoft, Tulsa, OK, USA). Two-way analysis of variance (ANOVA) for repeated measurements with factors mode and time were used for data analysis. Student-Newman-Keuls' post hoc test was used for comparison of significant ANOVA results within and between the groups. Data of the first measurement set (T_baseline_) were only compared with data of the second measurement set (T_0_). P values less than 0.05 were considered significant.

## Results

**Figure 2:**
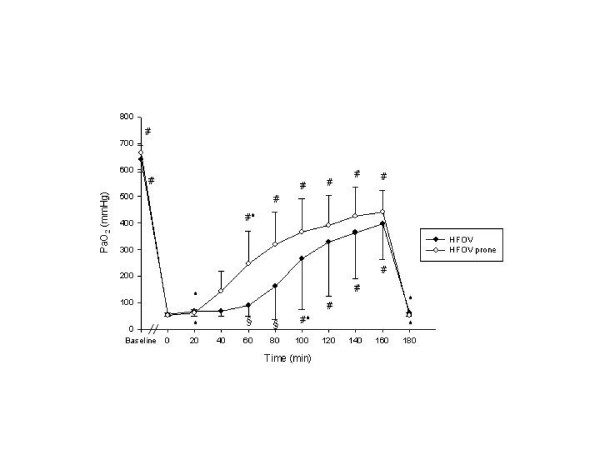
Arterial oxygen partial pressure (PaO_2_) throughout the study protocol Data are mean ± standard deviation. # p < 0.05 vs. T_0_; * p < 0.05 vs. T_T - 20_; § p < 0.05 HFOV vs. HFOV prone

**Figure 3:**
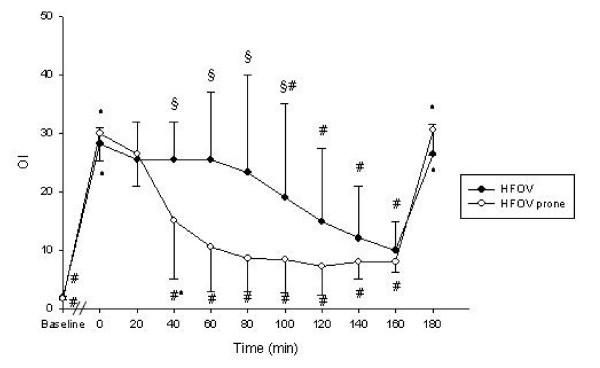
Oxygenation Index (OI) throughout the study protocol Data are mean ± standard deviation. # p < 0.05 vs. T_0_; * p < 0.05 vs. T_T - 20_; § p < 0.05 HFOV vs. HFOV prone

**Figure 4:**
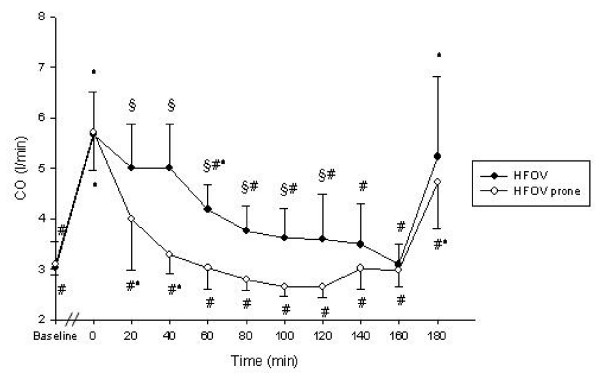
Cardiac output (CO) throughout the study protocol. Data are mean ± standard deviation. # p < 0.05 vs. T_0_; * p < 0.05 vs. T_T - 20_; § p < 0.05 HFOV vs. HFOV prone

Detailed data regarding hemodynamics, blood gases and respiratory parameters are presented in table 1. PaO_2_-, OI- and CO-changes during the experimental period are displayed in figures 2, 3, 4.

**Table 1:** Hemodynamic and metabolic data at baseline (T_baseline_), after injury (T_0_), after starting HFOV (T_20_), after randomisation (T_40_), during P_mean_-augmentation (T_60_-T_160_), at end of experiment (T_180_)

	Group	T_baseline_	T_0_	T_20_	T_40_	T_60_	T_80_	T_100_	T_120_	T_140_	T_160_	T_180_
PaCO_2_	HFOV	41 +/- 3 #	76 +/- 12 *	73 +/- 8	73 +/- 8	74 +/- 9	74 +/- 10	78 +/- 12	80 +/- 12	82 +/- 12	83 +/- 13	85 +/- 10 #
[mmHg]	HFOV prone	41 +/- 3 #	77 +/- 6 *	79 +/- 6	70 +/- 6	71 +/- 6	72 +/- 6	75 +/- 4	76 +/- 4	77 +/- 5	77 +/- 6	78 +/- 5
pH	HFOV	7,43 +/- 0,05 #	7,11 +/- 0,13 *§	7,15 +/- 0,09	7,15 +/- 0,09	7,19 +/- 0,1	7,21 +/- 0,09 #	7,21 +/- 0,1 #	7,15 +/- 0,13	7,15 +/- 0,13	7,15 +/- 0,13	7,16 +/- 0,12
	HFOV prone	7,45 +/- 0,03 #	7,22 +/- 0,1 *	7,19 +/- 0,09	7,2 +/- 0,06	7,23 +/- 0,06	7,21 +/- 0,05	7,21 +/- 0,05	7,19 +/- 0,06	7,18 +/- 0,07	7,19 +/- 0,07	7,22 +/- 0,1
Qs/Qt	HFOV	0,01 +/- 0,03 #	0,65 +/- 0,15 *	0,57 +/- 0,12	0,57 +/- 0,12 §	0,5 +/- 0,16 #§	0,42 +/- 0,21 *#§	0,33 +/- 0,26 *#§	0,24 +/- 0,2 *#	0,19 +/- 0,15 #	0,15 +/- 0,1 #	0,64 +/- 0,09 *
(ratio)	HFOV prone	0 +/- 0,02 #	0,56 +/- 0,07*	0,5 +/- 0,08	0,36 +/- 0,11 *#	0,24 +/- 0,13 #*	0,2 +/- 0,1 #	0,16 +/- 0,1 #	0,15 +/- 0,1 #	0,13 +/- 0,09 #	0,12 +/- 0,06 #	0,56 +/- 0,05 *
SgvO_2_	HFOV	79 +/- 10 #	63 +/- 10 *§	57 +/- 8	57 +/- 8	61 +/- 11	65 +/- 11	69 +/- 7	72 +/- 3	75 +/- 2 #	76 +/- 3 #	53 +/- 11 #*
[%]	HFOV prone	87 +/- 3 #	48 +/- 10 *	53 +/- 8	65 +/- 14 *#	67 +/- 5 #	73 +/- 4 #	71 +/- 6 #	72 +/- 7 #	74 +/- 3 #	75 +/- 4 #	47 +/- 11 *
PIP	HFOV	20 +/- 3 #	28 +/- 3 *	20 +/- 1 *#	20 +/- 1 #	23 +/- 1 *#	26 +/- 1 *#	28 +/- 1 *	32 +/- 1 *#	35 +/- 1 *#	37 +/- 1 *#	27 +/- 3 *§
[cmH_2_O]	HFOV prone	18 +/- 3 #	28 +/- 2 *	20 +/- 1 *#	20 +/- #	23 +/- 1 *#	26 +/- 1 *	28 +/- 1	32 +/- 1 *#	35 +/- 1 *#	37 +/- 1 *#	29 +/- 2 *
HR	HFOV	87 +/- 19	89 +/- 14 §	79 +/- 15	80 +/- 15 §	78 +/- 18 §	75 +/- 18 §	78 +/- 21 §	75 +/- 19 §	69 +/- 12 #	76 +/- 21	77 +/- 18
[/min]	HFOV prone	83 +/- 15 #	66 +/- 13 *	68 +/- 15	58 +/- 8	57 +/- 8	53 +/- 4	52 +/- 5	56 +/- 4	57 +/- 4	62 +/- 9	75 +/- 11
MAP	HFOV	79 +/- 13	89 +/- 11	81 +/- 12	81 +/- 12	81 +/- 8	85 +/- 8	84 +/- 5	84 +/- 5	82 +/- 5	84 +/- 9	84 +/- 4
[mmHg]	HFOV prone	83 +/- 9	84 +/- 7	83 +/- 7	83 +/- 10	81 +/- 8	79 +/- 8	80 +/- 7	85 +/- 12	78 +/- 5	78 +/- 8	83 +/- 7
MPAP	HFOV	23 +/- 5 #	32 +/- 6 *	33 +/- 6	33 +/- 6	33 +/- 6	34 +/- 5	37 +/- 6	37 +/- 4 #	38 +/- 4 #	39 +/- 4 #	33 +/- 5
[mmHg]	HFOV prone	22 +/- 6 #	29 +/- 4 *	34 +/- 4	32 +/- 6	35 +/- 5 #	34 +/- 3	35 +/- 3 #	35 +/- 4 #	36 +/- 2 #	36 +/- 2 #	30 +/- 4 *
CVP	HFOV	6,7 +/- 1,3 #	9 +/- 2,4 *	9,7 +/- 2,5	9,7 +/- 2,5	10 +/- 2,8 §	11,6 +/- 2,9 #	12 +/- 2,4 #	13,7 +/- 1 #	13,9 +/- 0,9 #	14,3 +/- 1,1 #	9,4 +/- 1,3 *
[mmHg]	HFOV prone	6,9 +/- 1,3 #	10,9 +/- 3,3 *	10 +/- 1	10,9 +/- 1,5	12,7 +/- 2,9	11,9 +/- 2,3	12,4 +/- 2,4	12,3 +/- 2,6	12,6 +/- 2,1	12,6 +/- 2,1	10,7 +/- 2,3
PCWP	HFOV	9,1 +/- 2,3	10 +/- 3,7	11 +/- 3,8	11 +/- 3,8	12 +/- 3,2	12,1 +/- 2,6	12,4 +/- 2	14,1 +/- 2,4 #	15 +/- 1,9 #	15 +/- 1,8 #	11 +/- 3,3 *
[mmHg]	HFOV prone	9,1 +/- 2,3	11,1 +/- 1,9	11,9 +/- 1,3	11,3 +/- 2,1	12,9 +/- 2,7	12,6 +/- 1,7	12,9 +/- 1,9	12,1 +/- 1,6	12,9 +/- 1,2	12,6 +/- 1,1	11,1 +/- 1,3

Data are mean ± standard deviation; Two-way-ANOVA with repeated measurements (Student-Newman-Keuls Method – post hoc test): * p < 0.05 vs. T_T-20_; # p < 0.05 vs. T_0_; § p < 0.05 HFOV vs. HFOV prone.

CVP = central venous pressure; HFOV = high frequency oscillatory ventilation; HR = heart rate; MAP = mean arterial pressure; MPAP = mean pulmonary artery pressure; PCV = pressure controlled ventilation; PCWP = pulmonary capillary wedge pressure; Qs/Qt = pulmonary shunt fraction; SgvO_2 _= mixed venous oxygen saturation.

### Lung Injury

All animals passing T_baseline _survived the study period. Acute lung injury was induced in all animals by means of repeated lung lavages (19 ± 2) with significant changes (p < 0.05) in PaO_2_, PaCO_2_, SvO_2_, P_mean_, PIP, Qs/Qt, CVP, MPAP and CO: PaO_2 _decreased from 652 ± 37.5 mmHg to 55 ± 7.5 mmHg, PaCO_2 _increased from 41 ± 3 mmHg to 76.5 ± 9 mmHg, SvO_2 _decreased from 83 ± 6.5 % to 60.5 ± 10 %, P_mean _increased from 11.5 ± 1.4 cmH_2_O to 16.5 ± 1.2 cmH_2_O, PIP increased from 19 ± 3 cmH_2_O to 28 ± 2.5 cmH_2_O, Qs/Qt increased from 1 ± 0.5 % to 60.5 ± 11 %, CVP increased from 7.8 ± 1.3 mmHg to 10 ± 2.9 mmHg, MPAP increased from 22.5 ± 5.5 to 30.5 ± 5 mmHg, CO increased from 3.1 ± 0.4 l/min to 5.7 ± 0.8 l/min. No significant differences could be detected between the 2 groups for the parameters tested at time points T_baseline _and T_0_. Reduction of P_mean _to the T_0_-level (T_180_) combined with PCV at T_0_-ventilator settings resulted in immediate significant increases in CO and Qs/Qt to T_0_-values in both groups without significant differences between the groups. Accordingly PaO_2 _decreased significantly to T_0_-levels.

### Pulmonary gas exchange

Oxygenation improved significantly with rising P_mean _in both groups. At T_80 _and T_100 _PaO_2 _was significantly higher in the prone positioned animals. A significantly higher PaO_2 _compared to the preceeding time point was detected at T_80 _in the prone positioned animals and at T_120 _in the animals positioned supine. Significant improvement of OI occurred immediately after prone positioning (T_40_) lasting until T_100_. SvO_2 _was significantly higher from T_40 _onwards in the HFOV-prone group if compared to T_0 _without detectable significant differences between the groups. All animals remained hypercapnic with a PaCO_2 _greater 70 mmHg resulting in a pH of less than 7.23 throughout the experiment in both groups.

### Respiratory parameters

PIP increased significantly in all groups with rising P_mean _without differences between the groups.

### Hemodynamics

MAP remained stable in both groups. CVP and PCWP started to rise significantly in the HFOV group from T_100 _and T_140 _respectively if compared to T_0_. MPAP was increased significantly in both groups from T_140 _if compared to T_0_. From T_40 _to T_140 _CO and HR were significantly lower and continuously falling in the HFOV-prone group. At T_160 _and T_180 _no differences between the groups could be detected regarding CO and HR. Qs/Qt was significantly lower from T_40 _to T_120 _in the HFOV-prone group without differences between the groups from T_140 _onwards.

## Discussion

We evaluated the effects of prone positioning combination of HFOV and prone positioning in an adult animal model of ARDS. The major findings of our study are: 1) HFOV and prone positioning improves oxygenation at a lower P_mean _than HFOV and supine positioning. 2) HFOV and prone positioning result in significant reduction of pulmonary shunt fraction and normalisation of cardiac output at a lower P_mean _than HFOV and supine positioning. 3) Hypercapnia was neither ameliorated by HFOV nor combination of HFOV with prone positioning.

Since therapeutic alternatives are lacking and the underlying concepts sound reasonable, multimodal therapeutic approaches are commonly used for salvage therapy in patients with ARDS [19]. Apart from subsets of patients in other HFOV trails, the combined use of HFOV and prone positioning is described in one case report and was investigated systematically in a prospective randomized study including 39 medical patients [15,20]. Papazian et al. found the prone position combined with HFOV and PCV superior to HFOV and supine positioning in terms of oxygenation, but failed to demonstrate additive effects. However, the inflammatory mediators were elevated during HFOV-prone but not during HFOV-supine. The authors themselves put these results into perspective, since a control group was lacking and a time dependent natural change in the concentration of inflammatory mediators could not be excluded. It is a limitation of our study, that we did not investigate a control group ventilated in a conventional lung protective mode and positioned prone in order to detect additive effects of the two treatment modalities. Papazian et al. stressed the difficulties associated with bronchoalveolar lavages in ARDS patients in terms of patient safety and feasibility. This calls for long term experiments with large animals comparing conventional lung protective ventilation and HFOV with and without prone positioning looking not only at gas exchange and respiratory mechanics but also at histology and inflammatory mediators.

Current concepts to ameliorate the detrimental effects of VILI focus on reduction of volutrauma, barotrauma, atelectrauma and biotrauma [21]. It was shown in a small animal model, that HFOV had the same effect on oxygenation and pulmonary compliance than a conventional lung protective ventilatory approach but also reduced the systemic inflammatory response [22-24]. However, tracheal tube size, respiratory frequency and pressure amplitude are markedly different in small animals resulting in non-comparable changes of pulmonary mechanics and oscillatory pressure transmission. Therefore, experiments in large animals should be performed before HFOV is assessed systematically in adult patients with ARDS. Aiming to simulate a life-threatening clinical scenario, we induced an acute lung injury with severe hypoxemia and hypercapnia.

It was striking, that 19 ± 2 lavages with 30 ml/kg isotonic saline were needed to reach the targeted PaO_2_-value, suggesting a lung protective effect of the low-tidal-volume approach during ARDS-induction even on a low PEEP-level (5 cmH_2_O). Intrinsic PEEP was measured during PCV by means of an endexpiratory occlusion maneuver for five seconds after every third lavage and was always less than 1 cm H_2_O. In two studies using sheep with a body weight of 30 kg, 4 lavages were needed to achieve a PaO_2 _of less than 120 mmHg. These animals were ventilated with a V_T _of 12 ml/kg in a volume controlled mode [25,26]. Stability of the experimentally induced ARDS was proven two-fold: 1) A stabilisation period of 60 min. with unchanged ventilatory parameters was kept between the last pulmonary lavage and T_0_. 2) After T_160_, HFOV and prone positioning were discontinued, all animals were ventilated with PCV and PEEP was reduced to 5 cmH_2_O [26]. This manoeuvre resulted in immediate reversal of PaO_2_, PaCO_2 _and hemodynamics to T_0_-values.

Although the combined application of HFOV and prone positioning improved oxygenation, normalized cardiac output and significantly reduced pulmonary shunt fraction at a lower P_mean _than HFOV alone, hypercapnia was not influenced in our experiment. This is consistent with clinical results, since normocapnia was not achievable with HFOV alone in many adults with ARDS [8,27]. The ability to control the PaCO_2 _with the least possible ventilator pressure amplitude, e.g. by using HFOV, might result in further lung protection [28]. However, V_T_-reduction increases the risk of hypercapnia, thereby aggravating the pulmonary inflammatory response [29]. Even though permissive hypercapnia does not increase mortality and might have beneficial effects, such as lung protection from reperfusion injury [30], there are clinical situations where hypercapnia is contraindicated [31]. We knew from pilot experiments that CO_2_-elimination could only be increased in our animal model using a respiratory rate of less than 3 Hz, losing the advantage of oscillation in reducing lung damage [32]. Another possibility to improve CO_2_-elimination during HFOV is deflation of the cuff of the endotracheal tube. Since comparability of P_mean _was a prerequisite for the study and airway-pressure measurement at the tip of the endotracheal tube are prone to artefacts, we could not realize this option. There might be a need to combine HFOV with extracorporeal CO_2_-elimination whenever normocapnia is mandatory, e.g. in patients with cerebral oedema [33].

A lung volume between the lower and upper inflection point, derived from the inflation pressure-volume curve, is traditionally interpreted as ideal for oxygenation [34]. Evidence is raising, that best oxygenation is a better indicator for an open lung-PEEP during a decremental PEEP-trial after a recruitment manoeuvre [35]. We aimed to achieve optimal lung volume using increases in PaO_2 _as a crude surrogate for alveolar recruitment and avoidance of hyperinflation. Since an initial recruitment manoeuvre in our animals might have resulted in fatal cardiovascular collapse and barotrauma [23], we increased P_mean _stepwise and did not create a pressure volume relationship [36,37]. Generation of a pressure volume curve can cause lung derecruitment [38]. Further deterioration of oxygenation in our animals would have implied a high risk of irreversible hypoxia. The severity of hypoxemia present in our animals after ARDS-induction was our motivation not to randomize the airway pressure changes but to stepwise increase P_mean_[39].

It is the major limitation of this study, that it was performed in pigs and not in patients. In adults not surfactant deficiency but alveolar flooding is the predominant mechanism in ARDS-development. It limits the transferability of most ARDS model derived results to clinical practice [40].

## Conclusion

In this saline lavage induced porcine model of ARDS, we showed in a clinically relevant scenario, that the combination of HFOV and prone positioning improved oxygenation at a lower P_mean _than HFOV combined with supine positioning. In addition, reduction of the pulmonary shunt fraction and normalisation of the cardiac output was achieved at lower airway pressures. The ventilator pressure amplitude is a major determinant of VILI. HFOV might be a step towards further lung protection, since sufficient oxygenation can be restored or maintained with a significant reduction of the ventilator pressure amplitude when compared to standard respirator modes. However, HFOV failed to be a major component in ARDS treatment algorithms in adult patients. Having in view a long history of failed multimodal treatment approaches in ARDS research, we now conclude from our results that a combination of HFOV and prone positioning seems promising and should be further investigated systematically and compared to conventional lung protective ventilation. Long term trials in large animals and aquisition of histologic and immunologic data clearly seem justified.

## Abbreviations

ARDS acute respiratory distress syndrome

CaO_2 _arterial oxygen content

CCO_2 _pulmonary capillary oxygen content

CDP continuous distending pressure

CO cardiac output

CvO_2 _mixed venous oxygen content

CVP central venous pressure

FIO_2 _fraction of inspired oxygen

HFOV high frequency oscillatory ventilation

HR heart rate

I:E inspiratory to expiratory ratio

MAP mean arterial pressure

MPAP mean pulmonary artery pressure

OI Oxygenation index

PaCO_2 _Arterial carbondioxide partial pressure

PaO_2 _Arterial oxygen partial pressure

PEEP positive end-expiratory pressure

PIP peak inspiratory pressure

PCV pressure controlled ventilation

PCWP pulmonary capillary wedge pressure

P_mean _mean airway pressure

Qs/Qt pulmonary shunt fraction

RR; f respiratory rate

V_T _tidal volume

## Competing interests

The author(s) declare that they have no competing interests.

## Authors' contributions

JB designed and coordinated the study and drafted the manuscript. JB and RM performed the experiments. MK performed the statistical analysis. MA, CG and NR participated in the design and coordination of the study and helped to draft the manuscript. All authors read and approved the final manuscript.

## Pre-publication history

The pre-publication history for this paper can be accessed here:


